# Neurobehavioral responses in swiss albino mice induced by an aqueous leaf extract from a medicinal plant named Heliotropium incanum Ruiz & Pav.

**DOI:** 10.6026/97320630016679

**Published:** 2020-09-30

**Authors:** H Ashalata Singha, Mahuya Sengupta, Meenakshi Bawari

**Affiliations:** 1 Assam University, Silchar, Assam, India 788011

**Keywords:** neurobehavioral changes, medicinal plant, anxiogenic, Heliotropium incanum, toxic effect

## Abstract

It is of interest to examine the adverse neuro-behavioural responses on mice treated with the aqueous crude extract of Heliotropium incanum (AEHI), which were evaluated using various behavioral paradigms. On the basis of median lethal dose value, doses of AEHI were chosen to be 150mg/kg and 440mg/kg for further experiment. Four groups comprising of five mice each were divided for the 14 days experiment. Group I, the control group, received distilled water; Group II and III received AEHI (150 mg/kg body weight and 440 mg/kg body weight) respectively; Group IV received standard drugs, Diazepam/Fluoxetine, administered orally. On administration of AEHI, it was revealed that dose 440 mg/kg showed less exploration activity in the hole board test; decrease in the number of squares crossed in locomotory test, time period in the open arm in the plus maze test was significantly reduced and the immobility time was significantly extended in comparison to control and standard drugs. The microscopic study of brain revealed damaged hippocampus along with nerve cells degeneration. Consequently, the results concluded that the outcome of the AEHI produced evidences for the anxiogenic activity in mice.

## Background

India possesses vibrant knowledge in ethnomedicinal plants which serve as medicines for curing various ailments [1]. The traditional medicine probably in the form of herb or crude drug is a complex mixture of compounds, some useful and some harmful, but all are amalgamated into a single chemical concoction [2,3]. Consequently, it perhaps alters the actions of the nervous system, as different phytochemical compounds are present in plants. Neurobehavioral effects play a great role in depicting the transformation of nerve cell communication and its integration and also reflect the morphological alterations, which can be measured through histological study. There are various behavioral tests designed to detect the dysfunction of the brain in mice [4]. These paradigms are associated to analyze the locomotory, sensory, exploratory and depressive functions. Four paradigms are considered in this study: elevated plus maze test (EPMT) in which the anxiety level is measured, hole board test (HBT) to examine the exploration behavior, forced swim test (FST) for assessing depression in mice and locomotor activity test (LAT) to measure the motor function. Heliotropium incanum Ruiz & Pav. (former name - Heliotropium indicum L.) belonging to the family- Boraginaceae is an annual herb commonly known as the "Cock's comb or Indian heliotrope" and locally called "Hathi soor". It has been used in different traditional practice of ailments such as skin diseases, stomachache, in whooping cough, in malaria, abdominal pain as well as act as an antitumor agent [5]. Contradictory to the medicinal properties, it has been reported that the plant extracts contain several alkaloids which showed hepatotoxicity [6-7]. In addition, it has been reported that pyrrolizidine alkaloid present in Heliotropium species exerted demyelination of the axons of the cerebral cortex [9]. Therefore, it is of interest to evaluate the adverse effect of aqueous crude extract of Heliotropium incanum Ruiz & Pav. (AEHI) emphasizing on the neurobehavioral responses displayed by mice using various neurobehavioral paradigms.

## Materials and methods:

The aerial parts of the plant Heliotropium incanum Ruiz & Pav were collected during their respective season from Tolengram (24.7987° N, 93.0207° E), Malugram (24.8415° N, 92.8094° E) of Cachar district, Assam, India. Identification and authentication were done by Botanical Survey of India, Shillong, Meghalaya, India bearing Identification no. - BSI/ERC/Tech//Identification/2016/314 and voucher specimen number Ph.D/AUS/ASHI/20. The name of the plant has been checked in the http://www.ipni.org/ipni/idPlantNameSearch.do?id=116935-1. Extraction of plant samples were done by the method described by [10].

## Determination of median lethal dose (LD50):

Adult mice (25-30g; 8-9 weeks old, n=4) of either sex were used for acute toxic study. Mice were fed standard pellet diet and had access to water ad libitum. Experimental protocols used in this study were conducted in accordance with Organization for Economic Co-operation and Development (OECD) guidelines and were approved by Institutional Animal Ethics Committee, Assam University, Silchar, India (Regn. No. -AUS/IAEC/2017/PC/07). They were randomly divided into six groups with four mice each. The control being in the first group received saline water whereas groups 2-5 were treated orally with aqueous extract of Heliotropium incanum at the doses of 800, 1200,1600, 2000,3000 mg/kg body weight (b.wt.) respectively. Food and water were provided immediately after the treatment. Mice were observed for behavioral changes and mortality for a period of 48 hours. The acute lethal dose was calculated using SPSS version 19.0 for probit analysis [11,12].

## Determination of the neurotoxicity:

Twenty adult Swiss albino mice (aged 8-9 weeks; 25-30g; n=5) are grouped independently into four containing five mice each. The first group as control received saline water, AQHI (150 mg/kg) was considered as second group whereas AQHI (440 mg/kg) as the third and Diazepam / Fluoxetine as the fourth group, administered orally by feeding needle for 14 days. On the 15th day, both control and treated mice were assessed for their neurobehavioral assessments. Hole board test (HBT) [13]: The hole board apparatus consists of a wooden chamber (40x40x40 cm3) with four equidistant holes, having 3 cm diameter. Each hole was distributed evenly on the floor elevating to a height of 25 cm from the ground. The mice were placed in the apparatus after the treatment for 30 minute. The number of head poking was recorded for 20 min with intervals of 5 min. Locomotor activity test (LAT) [14]: Mice were tested in a wooden box (45 cmx25 cm) divided into 16 squares. The number of squares travelled was counted for 20 min with an interval of 5 min per mouse. Elevated plus maze test (EPMT) [15]: The apparatus consist of two closed arms crossed with two open arms (35x5x20 cm3). A square (5x5 cm2) in the centre connect the arms. The height of the arms from the ground is elevated to 25 cm. After 30 min of treatment, the mice were placed in the center facing the closed arm. The duration spent in both the arms was recorded. An entry is described when all the four paws are inside the arms. Forced swim test (FST) [16]: Here, Plexiglas cylinder (10 cm diameter, 50 cm height) was used, filled with 10 cm high room temperature water. Mice were placed in order to measure the immobility time for 20 min with a break of 5 min per mouse. Here, immobility denotes when it remained float in the water with head above water.

## Histo-pathological study:

After the final exposure, the animals were deprived of food for 16 h and decapacitated to remove the brain. It was then fixed in 10% buffered formalin and further processed for histological examination. Sections of tissues embedded in paraffin wax were obtained with the thickness of 5µm and stained with haematoxylin and eosin and then observed under light microscope.

## Statistical analysis:

Data are presented as mean ± S.E.M. The one -way analysis of variance (ANOVA) is conducted followed by Tukey's post hoc multiple comparison test with Bonferroni correction to reduce the dominance of type-I error problem. Probability level at p<0.05 as significance was used.

## Results:

### Acute toxicity (LD_50_)

**Figure 1:**
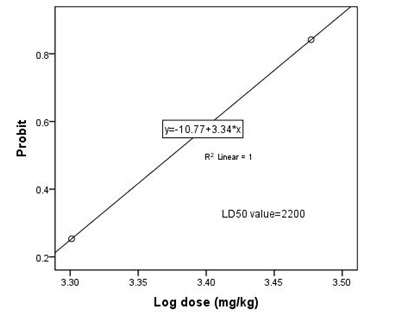
Determination of LD50 value for the aqueous extract of the leaves of Heliotropium incanum orally administered to mice using SPSS probit analysis. Log dose=3.342=2200 mg/kg

Mice showed an increased dormancy, the consumption of water and food reduced, restlessness also can be seen before death. Mortality of mice was observed from the dose 1600 mg/kg b.wt. to 3000 mg/kg bwt. Therefore, LD50 of AQHI was calculated to be 2200.00 mg/kg b.wt. (Table 1 - see PDF and Figure 1)

### Preliminary phytochemical screening:

Based on the investigations of the aqueous extract of Heliotropium incanum, the various phytoconstituents present in the aqueous extract of AEHI are alkaloids, phenolic compounds, saponins, flavonoids, tannins, protein and amino acids (Table 2 - see PDF).

### Neurobehavioral study:

**Figure 2:**
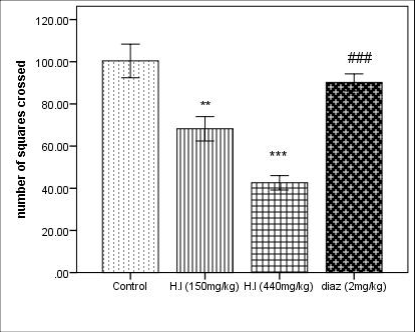
Time spent in open arms in elevated plus maze. Each column represents mean ± SEM (n=5). Comparisons were made using ANOVA followed by Tukey's multiple comparison test with Bonferroni correction where "***" denotes comparison between AEHI (440 mg/kg) and control, p<0.001 and "###" denotes comparison between diazepam and AEHI (440 mg/kg), p<0.001

**Figure 3:**
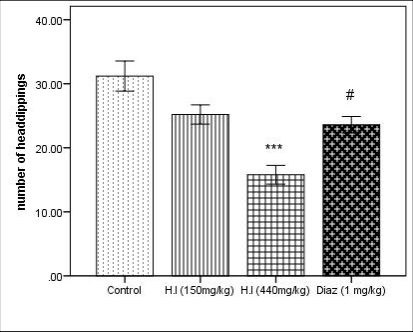
Number of head dippings in the hole board test. Each column represents mean ± SEM (n=5). Comparisons were made using ANOVA followed by Tukey's multiple comparison test with Bonferroni correction where "***" denotes comparison between AEHI (440 mg/kg) and control, p<0.001 and "#" denotes diazepam vs AEHI (440 mg/kg), p<0.05

**Figure 4:**
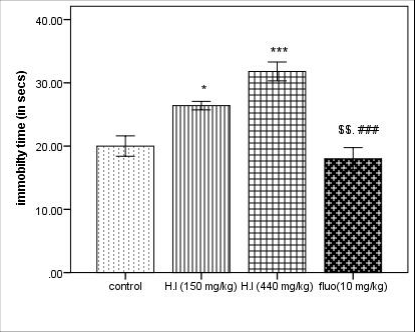
Immobility time in the forced swimming test. Each column represents mean ± SEM (n=5). Comparisons were made using ANOVA followed by Tukey's multiple comparison test with Bonferroni correction where "*" denotes AEHI (150 mg/kg) vs control, p<0.05; "***" denotes comparison between AEHI (440 mg/kg) and control, p<0.001 and "$$" denotes fluoxetine vs AEHI (150 mg/kg), p<0.01; "###" signifies the comparison between 440 mg/kg AEHI and fluoxetine p<0.001.

**Figure 5:**
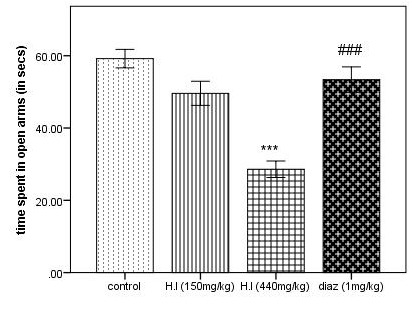
Number of squares crossed in the locomotor activity test. Each column represents mean ± SEM (n=5). Comparisons were made using ANOVA followed by Tukey's multiple comparison test with Bonferroni correction where "**" denotes AEHI (150 mg/kg) vs control, p<0.01; "***" denotes comparison between AEHI (440 mg/kg) and control and "###" denotes diazepam vs AEHI (440 mg/kg), p<0.001

Mice treated with the dose 440 mg/kg of AEHI showed significant decrease in the time spent in the open arms (p<0.001) as compared with the control, as observed in the Figure 2. However, a significant difference is seen between the standard drug and AEHI (440 mg/kg) implying that the plant extract did not show anxiolytic activity. In Figure 3, there is a significant decrease in the head poking when treated with AEHI (440 mg/kg, p<0.001) while comparing with the control group in the HBT. On the other hand, standard drug, Diazepam showed significant increase when compared with 440 mg/kg dose of AEHI. As shown in Figure 4, the immobility time is raised more significantly in the mice treated with AEHI (150 mg/kg, p<0.05; 440 mg/kg, p<0.001) as compared with the control group. However, fluoxetine produced a significant decrease in immobility time in this test and showed that the dose of the plant extract failed to modify the immobility time as compared to standard drug. The activity in locomotion is seen in Figure 5 where the number of squares crossed by the AEHI treated mice of dose 150 mg/kg and 440 mg/kg are significantly minimized as compared to control, p<0.01 and p<0.001 respectively. However, increase in the number of squares crossed is observed in the treated mice comparing with a standard drug.

### Histopathological study:

**Figure 6:**
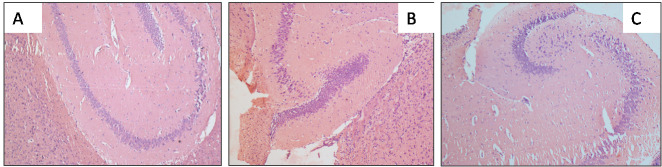
Microscopic study of hippocampus in mouse brain. Grossly (x100). Histological sections of brain were stained with hematoxylin & eosin (H&E). Control (A) shows normal nerve cells. Exposed mice to 150 mg/kg/day AEHI (B) shows moderate scattered of neuronal cells. Treated mice with 440 mg/kg AEHI (C) shows extreme damage.

In the present study, brain tissues were prepared from control, two different doses (150 mg/kg and 440 mg/kg b.wt.) of AEHI respectively. Changes were observed in the brain of HI treated mice compared to control. Figure 6 shows the hippocampal area of cerebral cortex after staining with haematoxylin and eosin. Control mice (Figure 5A) demonstrated the normal intact structure of hippocampus. In Figure 5B, a minor lesion can be seen whereas in Figure 5C massive cellular depletion, neurofibrillary degeneration is noticed.

## Discussion:

The present study aimed to investigate the neurobehavioral effects of the different doses (150mg/kg and 440mg/kg b.wt.) of aqueous crude extract of Heliotropium incanum on mice. In the result, both the doses (150 mg/kg and 440 mg/kg b.wt.) induced neurotoxicity in the cerebral cortex, however, high significant differences between the control and AEHI of dose 440 mg/kg were observed in locomotor activity test, suggesting that the treatment induced by AEHI causes motor abnormalities. This effect is found opposite to the effect observed after the reference standard drug, diazepam. Locomotion shows the sign of activeness; hence, there is a sign of sedative activity when it decreases [17]. The impairments of exploratory behaviors in the hole-board test are also found. The dose 440 mg/kg significantly decreased the dipping of head as compared to control and standard drug, Diazepam. Often, head dippings of mice are studied since it indicates explorative behavior. The decreased head poking implies the anxiogenic state of animals implicating that there might be various neurotransmitters which help in the behavior of exploration [18]. The data also indicate that the HI-induced mice showed immobility in forced swim test. This test has been accepted as a model of depressive-like behavior in rodents [19]. The immobilization time expanded significantly which indicates the hopeless, depressant activity [20] of the plant extract which is opposite to the effect shown by control group and the antidepressant drug, Fluoxetine. In elevated plus maze test, there was significant depletion of time spent in open arms. The less exposure period of mice to an elevated open maze indicates anxiety and fearfulness [21]. Studies had shown that stress produces behavioral alterations [22].

Previous studies have shown that the seeds of Heliotropium incanum contained pyrrolizidine alkaloids, heliotrine and its derivatives which exert hepatotoxic by forming tumors [23]. In addition, the ganglion blocking activity had also been reported due to heliotrine compound via reducing the stimulation of neurotransmitter which leads to demyelination of axons [24]. Studies have reported that flavonoids present in the plant could influence both anti-oxidant as well as pro-oxidant based on doses [25]. Hence, in this study, induction of neurotoxicity could be partly due to the presence of various phytoconstituents in the crude extract. Furthermore, hippocampal area of the brain is vulnerable to various stimuli and plays a major part in memory and exploration. We observed loss of neuronal density and distortion of cells in the regions of hippocampus induced by both doses (150 mg/kg and 440 mg/kg) of HI extract as compared to control. It has been reported that a deterioration in the hippocampal area leads to declination in learning and reduction in motor functions [14]. Therefore, it may be suggested that the plant crude extract have some toxic effect on the brain, which is the cause for neurobehavioral alterations.

## Conclusion

We provide evidence for the adverse effect of aqueous extract of Heliotropium incanum Ruiz & Pav. on mice. The results of various behavioral assessments indicated that the aqueous extract of H. incanum enhanced anxiety and impairment in motor performance. Distinct morphological alterations in the hippocampus also suggested that the extract induced marked loss of nerve cells in the brain. Thus, prolonged use of certain plants is necessary to examine its depressant mechanisms.
